# Malnutrition Imparts Worse Outcomes in Patients Admitted for Acute Pancreatitis

**DOI:** 10.7759/cureus.35822

**Published:** 2023-03-06

**Authors:** Alexander Le, Amjad Shaikh, Mohsin Ali, Ayham Khrais, Yazan Abboud

**Affiliations:** 1 Internal Medicine, Rutgers University New Jersey Medical School, Newark, USA

**Keywords:** immunology, alcohol, pancreatitis, nutrition, malnutrition, acute pancreatitis

## Abstract

Purpose

Cessation of enteral nutrition is usually a part of the early stage of acute pancreatitis (AP) treatment. To our knowledge, there is no large database study that examines the effects of preexisting malnutrition on the morbidities of patients admitted for acute pancreatitis. We aimed to investigate the effects of malnutrition on patients admitted for acute pancreatitis.

Methods

Data between 2008 and 2014 from the National Inpatient Sample (NIS) database was extracted. Inclusion criteria included patients with a primary diagnosis of AP using the International Classification of Diseases, Ninth Revision (ICD-9) codes, and ages greater than 17. Exclusion criteria included ICD-9 codes for chronic pancreatitis. The study group consisted of patients with a primary diagnosis of AP and a concurrent diagnosis of malnutrition. In-hospital mortality was compared using univariate and multivariate analyses to generate odds ratios. Elixhauser comorbidity scores predicting mortality and readmission were calculated based on weighted scores from 29 different comorbidities and compared using univariate analysis.

Results

Patients with malnutrition were significantly more likely to experience in-hospital mortality, sepsis, severe sepsis, septic shock, and respiratory failure. Malnutrition was found to increase mortality. Female sex and Black or Hispanic race showed lower mortality.

Conclusion

We hypothesize that there are likely other preexisting comorbidities that lead to malnutrition before the onset of pancreatitis. Malnutrition can cause impaired healing and the ability to recover from acute inflammation, which may be why the study group had a higher rate of sepsis.

## Introduction

Acute pancreatitis (AP) is an acute inflammatory process of the pancreas that typically involves a systemic inflammatory response that affects various organs. Diagnosis is made when two of three criteria are met: (1) abdominal pain consistent with pancreatitis, (2) serum lipase or amylase levels greater than three times the upper limit of normal, or (3) imaging consistent with pancreatitis [1]. In 2015, there were about 390,000 hospitalizations in the United States for AP, making it one of the most common gastrointestinal reasons for hospital admission [2-4]. While the United States healthcare system has improved in terms of access, imaging, and intervention, the burden of AP is predicted to increase. Similarly, mortality has remained relatively stable at 5-17% in severe AP and 1.5% in mild AP [5].

The three most common causes of AP are gallstone/biliary-related, alcohol-related, and idiopathic [5]. With that being said, one of the most common comorbidities of patients presenting with AP is malnutrition. Malnutrition as defined by Johns Hopkins Medicine is a condition where the body is deprived of vitamins, minerals, and nutrients necessary to maintain healthy tissue and organ function [6]. This includes those who are overnourished and undernourished. Overnutrition occurs in individuals who eat too much, don’t exercise enough, or consume too many vitamins. Undernutrition occurs in individuals who do not have enough essential nutrients for their daily requirements; this may be secondary to chronic alcoholism, malignancy, intestinal malabsorption, or decreased oral intake [7].

During episodes of AP, there are high nutritional requirements, and hypermetabolism increases the demand for vitamins B, C, and A and zinc among others due to the inflammatory process [8,9]. Severe inflammation leads to protein and lipid catabolism, increased insulin resistance, and impaired lipid clearance [8,9]. As a result, some patients may end up with acute necrotizing pancreatitis with 40-70% of patients developing sepsis with 80% mortality [10]. With this in mind, we seek to investigate the outcomes of patients with malnutrition who present with AP and sepsis.

## Materials and methods

Data/source population

Data between 2008 and 2014 from the Nationwide Inpatient Sample (NIS) database was extracted. Inclusion criteria for both groups included patients with a primary diagnosis of acute pancreatitis and malnutrition using the International Classification of Diseases, Ninth Revision (ICD-9) codes, and ages greater than 17. Exclusion criteria included all ICD-9 codes for chronic pancreatitis. The study group consisted of patients with a primary diagnosis of acute pancreatitis and a concurrent diagnosis of malnutrition. A case-controlled dataset was generated with cases controlled by age, gender, race, and median household income.

Statistical analysis

In-hospital mortality was compared between the two groups. Chi-square analysis was used for categorical variables and an independent samples t-test was used for continuous variables. The Elixhauser and Charleston comorbidity scores that predict mortality and readmission were calculated based on weighted scores from 29 different comorbidities. Scores were compared between the two groups using an independent sample t-test. Comorbidities such as myocardial infarction, congestive heart failure, peripheral vascular disease, cerebrovascular accident, dementia, a chronic obstructive pulmonary disorder, liver disease, diabetes mellitus, connective tissue disease, moderate to severe chronic kidney disease, solid tumor, leukemia, lymphoma, and acquired immunodeficiency syndrome were also examined between the groups. Multivariate analysis using binary logistic regression was conducted with death as a primary outcome. Primary outcomes measured included inpatient mortality. Secondary outcomes included length of stay, total charge during admission, and rates of sepsis, severe sepsis, and respiratory failure.

## Results

The initial dataset was generated before the case-control (Table 1).

**Table 1:** Baseline patient demographics SD: Standard deviation

	Acute Pancreatitis without Malnutrition (N=1849162)	Acute Pancreatitis with Malnutrition (N=102056)
Age - mean (SD)	51.13 (17.8)	55.62 (17.23)
Race		
White	1082633 (64.9%)	64104 (62.8%)
Black	279409 (16.7%)	14917 (14.6%)
Hispanic	211821 (12.7%)	7045 (7.7%)
Asian or Pacific Islander	31280 (1.9%)	1876 (2.1%)
Native American	14183 (0.8%)	703 (0.8%)
Other	49839 (3.0%)	2627 (2.9%)
Sex - no. (%)		
Male	965235 (52.2%)	51999 (51%)
Female	882525 (47.8%)	50051 (49%)

Across both groups, a majority of the patients were White. More than 50% of each group was also male. Case-controlled groups were generated with non-significant differences between the groups across all categories (Table 2).

**Table 2:** Case-controlled groups SD: Standard deviation

	No Malnutrition	Malnutrition
Age (SD)	56 (17)	56 (17)
Sex (%)		
Male	49743 (50.9)	50376 (50.9)
Female	48040 (49.1)	48684 (49.1)
Race		
White (%)	62887 (70.7)	63644 (70.6)
Black (%)	14365 (16.1)	14698 (16.3)
Hispanic (%)	6745 (7.6)	6960 (7.7)
Asian or Pacific Islander (%)	1807 (2.0)	1727 (1.9)
Native American (%)	642 (0.7)	601 (0.7)
Other	2562 (2.9)	2485 (2.8)
Median Income Quartile		
0th to 25th Percentile	31580 (32.3)	32073 (32.4)
26th to 50th Percentile	26932 (27.5)	27304 (27.6)
51st to 75th Percentile	22059 (22.6)	22285 (22.5)
76th to 100th Percentile	15603 (16)	15791 (15.9)

Patients with malnutrition were significantly more likely to experience in-hospital mortality, sepsis, severe sepsis, septic shock, and respiratory failure (Table 3).

**Table 3:** Univariate analysis of case-controlled groups

	No Malnutrition	Malnutrition	p-value
Death (%)	963 (1.0%)	3048 (3.1%)	<0.01
Sepsis (%)	887 (0.9%)	3640 (3.7%)	<0.01
Severe Sepsis (%)	672 (0.7%)	4103 (4.1%)	<0.01
Septic Shock (%)	396 (0.4%)	2477 (2.5%)	<0.01
Respiratory Failure (%)	1805 (1.8%)	9985 (10.1%)	<0.01
Length of Stay (SD)	5 (6)	11 (13)	<0.01
Total Charges Dollars (SD)	31938 (49978)	84102 (148305)	<0.01

**Figure 1:**
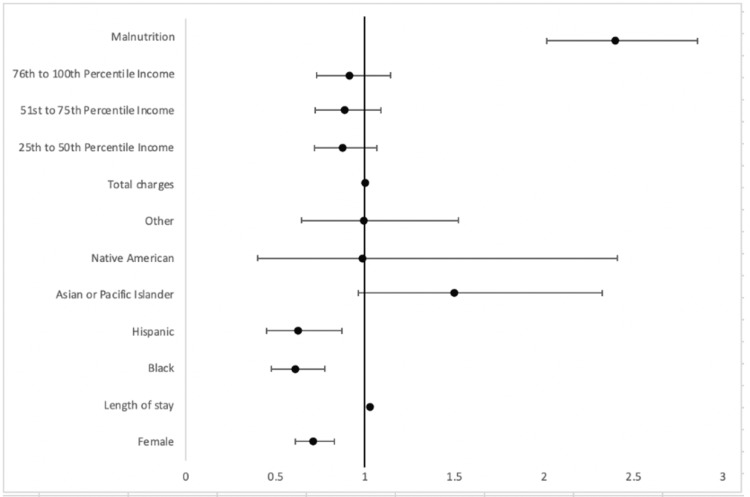
Multivariate analysis of inpatient mortality

On multivariate analysis (Figure 1), malnutrition was found to significantly increase mortality. Female sex and Black or Hispanic race were found to be associated with decreased mortality.

## Discussion

Our data suggest that malnutrition is associated with higher rates of death, sepsis, severe sepsis, septic shock, and respiratory failure. Furthermore, the female sex and Black or Hispanic race were found to be associated with decreased mortality. Nutrition is an often-complicated topic and difficult to assess clinically. A person’s nutritional status depends on not only their ingestion of macronutrient groups including carbohydrates, fats, and lipids but also essential micronutrients such as metals (zinc, copper, selenium, etc.) and vitamins (fat and water-soluble vitamins) [11]. Malnutrition can arise from the underconsumption or overconsumption of these previously stated nutrients and can be inhibited due to intake, malabsorption, and impaired metabolism [12]. This variability adds difficulty to developing a unified way of assessing a person’s nutritional status. Some clinicians use albumin and other lab markers as a proxy for nutritional health; however, a multitude of factors affect albumin levels and can lull clinicians into thinking a patient has better nutritional status than would be realized in history and physical [13].

A poor nutritional status may present as weight loss, decrease in muscle mass, loss of subcutaneous fat, and decreased strength or be present only subtly as changes in hair, skin, and nails and symptoms of fatigue, poor mobility, or increased healing times [14]. Moreover, poor nutritional status is associated with worse outcomes after discharge from the hospital including dependence and daily activities of living, nursing home requirements, and death within one year after discharge [15-20]. There are well-documented clinical associations with vitamin deficiencies and resulting symptoms such as poor wound healing [21].

Despite there being well-documented literature on the negative outcomes of malnutrition, there seems to be a lack of evidence showing that intense nutritional interventions play a role in reversing outcomes. Malnutrition can rise for a plethora of reasons including malignancy, gastrointestinal abnormalities/manipulation, gastrointestinal motility/absorption abnormalities, chronic illness (kidney, cardiac, pulmonary), autoimmune disease, and more [18,21].

Excessive alcohol use is a well-established and studied source of malnutrition in patients [22]. Alcohol can lead to hypoglycemia and poor carbohydrate metabolism as well as glucose intolerance. Alcohol can lead to increased protein turnover and result in a negative nitrogen balance. Lastly, alcohol alters lipid metabolism and leads to lipolysis [22]. Certain well-studied phenomena such as beer potomania describe electrolyte disturbances such as hyponatremia as a result of excessive alcohol consumption [22]. Patients with alcohol typically do not decrease their consumption of other foods to compensate for the calories that they ingested alcohol, but paradoxically they have a resultant decrease in their weight likely due to malabsorption and poor metabolism [22-24].

One of the proposed mechanisms of alcohol-altering energy metabolism is damage to the mitochondria which leads to increased thermogenesis and sympathetic nervous activation [23]. Furthermore, alcohol causes mucosal erosions and villous-predominant epithelial loss that can cause decreased absorption of vital nutrients; however, the pathophysiology of intestinal damage is poorly understood [23]. Other factors such as bacterial overgrowth, increased intestinal permeability, and alteration of intestinal motility can also contribute to malnutrition [22,23].

Alcohol remains the number one cause of acute pancreatitis in the United States [22]. Our study shows that patients that are diagnosed with malnutrition and then have an episode of acute pancreatitis have higher rates of morbidity and mortality, longer lengths of stay, and higher hospitalization costs. The exact mechanism underlying these poor outcomes is not well understood. Other causes of pancreatitis include gallstone pancreatitis, hypertriglyceridemia-induced pancreatitis, medications, and more. Pancreatitis causes patients to develop severe pain, decreased appetite, nausea, and vomiting [23].

Several articles implicate the role that nutrition plays in the function of the immune system as well as subsequent dysfunction with malnutrition [14,17,21,25]. Zinc, for example, has recently been shown to play a role in both the innate and adaptive immune response. Therefore, zinc deficiency leads to an impaired immune response [26]. Because the immune response is an energy and mineral-intensive process, both macro- and micro-nutrients have been implicated in the development of an appropriate response when the body comes into contact with pathogenic organisms. In particular, given that gut-associated immune tissue comes in contact with both normal gut flora and pathogenic organisms, it must regulate and mount a response fairly regularly and appropriately [14,21].

A few of the limitations of this study include the limitations of the NIS database and methods of coding for certain diagnoses. Given the difficulty with recognizing malnutrition clinically, it may have been underreported in the control group. Currently, there are a few assessment tools such as the Global Leadership Initiative on Malnutrition criteria, Nutritional Risk Screening, Geriatric Risk Index, and Nutritional Risk Screening 2002 with variable criteria including biochemical markers, anthropometry, mobility, cognitive state, and self-perception [12,27]. These tools also have non-uniform validity in the literature. NIS also does not code for the degree of malnutrition present to stratify the patients into high-severity or low-severity groups to identify whether severely malnourished patients have worse outcomes than moderately or mildly deficient patients. Furthermore, hypoalbuminemia is sometimes used as a surrogate for malnutrition and may be coded as such despite evidence to suggest that albumin may be affected by an inflammatory response in sick patients secondary to increased permeability and shifting and does not entirely correlate with clinical malnutrition. This may have affected the data in that a larger portion of patients with liver disease or a severe inflammatory response were included in the malnutrition group but did not suffer from malnutrition.

## Conclusions

Malnutrition has associations with poorer outcomes in patients hospitalized for acute pancreatitis and more instances of sepsis and septic shock. More studies need to be performed to elucidate the exact mechanism behind the impaired immune functioning due to malnutrition. Further studies need to be conducted to elucidate whether aggressive nutrition supplementation may play a role in improving outcomes in patients with acute pancreatitis. More research should be done to describe which limiting nutrients play the biggest role in a diminished immune response and how to best ameliorate the poor outcomes noticed in our study. Assessment and evaluation of malnutrition rely on a variety of assessment tools with different diagnostic criteria and uncertain evidence. Future efforts to obtain unifying criteria upon which to base the coding of a diagnosis of malnutrition may alert clinicians to intervene earlier.
